# Avian gyrovirus 2 in poultry, China, 2015–2016

**DOI:** 10.1038/emi.2016.113

**Published:** 2016-10-26

**Authors:** Shuai Yao, Tianbei Tuo, Xiang Gao, Chunyan Han, You Li, Yulong Gao, Yanping Zhang, Changjun Liu, Xiaole Qi, Honglei Gao, Yongqiang Wang, Xiaomei Wang

**Affiliations:** 1 Division of Avian Infectious Diseases, State Key Laboratory of Veterinary Biotechnology, Harbin Veterinary Research Institute, Chinese Academy of Agricultural Sciences, Harbin 150069, China; 2 Northeast Agricultural University, College of Veterinary Medicine, Harbin 150030, China; 3 Northeast Forestry University, College of Wildlife Resource, Harbin 150040, China

**Dear Editor**,

In 2015, a novel virus of the family *Circoviridae*, avian gyrovirus 2 (AGV2), was detected in chicken flocks and healthy humans in mainland China for the first time.^1^ AGV2, which was first detected by Rijsewijk *et al.*^2^ in early 2011, is a distant relative of chicken anemia virus (CAV), previously identified in diseased chickens in Brazil. During the same year, a human gyrovirus (HGyV) with a sequence that was highly homologous to that of AGV2 was detected on the skin of humans in France.^3^ Although the virus is poorly understood, Bullenkamp *et al.*^4^ found that the AGV2 VP3 protein, similar to CAV VP3, or apoptin, could induce tumor cell apoptosis. Increasing evidence has suggested that AGV2 infects both chickens and humans, which poses a great potential threat to not only the poultry industry but also human health. AGV2/HGyV DNA has been identified by polymerase chain reaction (PCR) in the blood of healthy humans, transplant patients and HIV-positive (positive for human immunodeficiency virus) patients.^5, 6^ AGV2 infections have furthermore been identified in diverse locations, such as Italy, South Africa, The United States, Brazil and The Netherlands, suggesting a worldwide distribution.^5, 7, 8^ Moreover, a study focusing on the detection of AGV2 as a contaminant of commercially available poultry vaccines identified the presence of AGV2 in 9 out of 32 live vaccines produced in Brazil, Canada and The Netherlands,^9^ which may be an explanation for the widespread distribution of AGV2. Thus, exploration of the AGV2 epidemic and AGV2 infections is warranted, especially in poultry, one of its primary hosts.

We performed an epidemiologic investigation of AGV2 with a study area that encompassed most parts of China from April 2015 to April 2016. Briefly, 448 poultry samples (282 in 2015 and 166 in 2016) were collected from 15 provinces. The samples comprised mostly liver, spleen and thymus samples obtained from clinically ill adult fowl and fowl embryos. Whole DNA was extracted from the samples and used as templates for specific PCR amplification with the AGV2-specific primers AGV2-F (5′-CGT GTC CGC CAG CAG AAA C-3′) and AGV2-R (5′-GGT AGA AGC CAA AGC GTC CAC-3′).^1^ We also detected other viruses using PCRs specific for avian reovirus (ARV), avian leukosis virus (ALV), Marek's disease virus (MDV), reticuloendotheliosis virus (REV), CAV and avian adenovirus (ADV).^10, 11, 12^ All positive PCR products were sequenced for verification.

Of the analyzed samples, 55 (12.28% 45 in 2015; 10 in 2016) were positive for AGV2, and these samples were obtained from locations distributed across 11 provinces. The number and percentages of positive samples by province are shown in Figure 1A. Forty-seven (85.45%) positive cases were identified in North China, and positive samples were obtained especially frequently in the northeast. The distribution implied that AGV2 is extensively present in mainland China. Fewer positive cases were detected in the southern region, probably because of the limited quantity of samples collected in that area; however, AGV2 may also be widely distributed in South China. Samples collected during autumn were more frequently AGV2-positive (March, April and May correspond to Spring; June, July and August correspond to Summer; September, October and November correspond to Autumn; December, January and February correspond to Winter.) than those collected during any other season. In winter and early spring, however, most AGV2-positive results were obtained in the warmer south of China (Figure 1b). This suggests that autumn might be a season of high occurrence because of optimal conditions for infection.

Monoinfection was detected in only 10 (18.19%) samples; the remaining (81.82%) samples indicated mixed infection. The 24 dual infections included AGV2 and the following coinfecting viruses: ARV (*n*=3), ALV-J (*n*=1), MDV (*n*=13), REV (*n*=4), CAV (*n*=2) and ADV (*n*=1). The 18 triple infections included AGV2 and REV+MDV (*n*=9), CAV+MDV (*n*=3), ADV+MDV (*n*=5) and ARV+MDV (*n*=1). Only three quadruple infections were identified; in these cases, AGV-2 was identified with CAV+REV+MDV (*n*=2) and ARV+ADV+MDV (*n*=1). Thus, mixed infections were the most frequent AGV2 infection type, and among the dual infections, coinfection with MDV was the most common (75.76%). MDV is a major pathogenic cause of chicken immunosuppressive disease; therefore, MDV infection might contribute to AGV2 pathogenesis.

There was only one AGV2-positive goose, whereas the only other AGV2-positive birds were chickens, including broilers and layers. It can therefore be inferred that chickens are one of the main hosts of AGV2; however, whether any chicken breeds are more commonly infected needs further investigation. Given that the chickens from which samples were obtained ranged from 10-day-old embryonated eggs to 240-day-old adults, further study of whether hosts of all ages are susceptible to this virus is necessary, as is determination of whether the virus is capable of vertical transmission.

Although no detailed symptoms of AGV2 infection have yet been reported, some novel clinical findings were observed at autopsy, including hemorrhage, edema, erosion in the glandular stomach, and swelling in the sciatic nerve, face and head. AGV2 has been detected in the brains of chickens with neurologic symptoms from flocks in South Africa, resulting in high mortality;^7^ therefore, the disease might be associated with digestive and neuronal tropism. AGV2 infection can cause lesions and even death in infected hosts. In one instance, a chicken flock infected with AGV2 but not any other detected pathogen displayed high daily rates of mortality (10 out of 80 chickens); however, the fact that no clinical symptoms or deaths were observed in ducks that were raised with these chickens suggests host specificity for AGV2.

In recent years, various other *Gyrovirus* (GyV) species have been discovered. Phan^13^ and Chu^14^ identified GyV3 and GyV4, respectively, in human feces. GyV5 and GyV6 were discovered in diarrheic samples from Tunisian children.^15^ AGV2 has public health significance because of its digestive and neurological symptoms and the risk of contamination through blood and feces. The pathogenesis of this virus in humans and chickens needs to be further investigated.

## Figures and Tables

**Figure 1:**
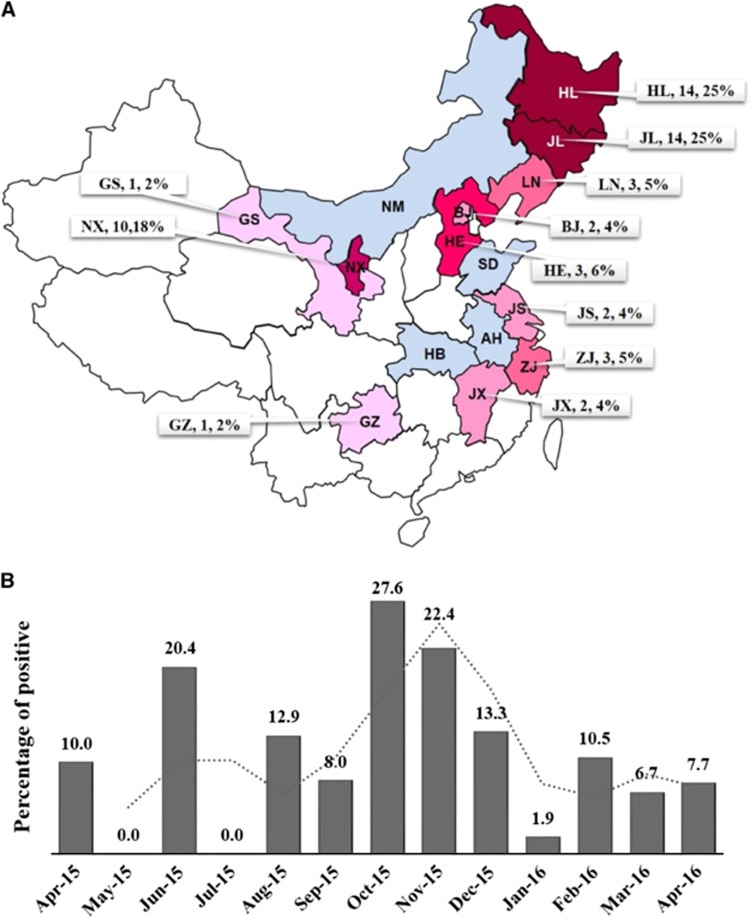
Temporal and spatial distribution of avian gyrovirus 2 China from April 2015 to April 2016. (**A**) The distribution of avian gyrovirus 2 (AGV2) in different provinces of China between April 2015 and April 2016. The labels show the name of the province and the number and percentage of AGV2-positive cases. The provinces colored in different shades of red indicate a positive result for AGV2. The darkness of the color indicates the percentage of AGV2-positive samples. The darkest red marks the highest value. Negative results are shown in blue, and blank regions indicate that no samples were collected. Anhui Province, AH; Beijing, BJ; Gansu Province, GS; Guizhou Province, GZ; Hubei Province, HB; Hebei Province, HE; Heilongjiang province, HL; Jilin province, JL; Jiangsu Province, JS; Jiangxi Province, JX; Liaoning province, LN; inner Mongolia autonomous region, NM; Ningxia Province, NX; Shandong Province, SD; Zhejiang Province, ZJ. (**B**) The curve of positive results depicting the change over time from April 2015 to April 2016. The percentage of AGV2-positive results per month is shown above the column, and an added trend-line shows the trend of the epidemic in a year.
